# Corn Rootworm: Biology, Ecology, Behavior, and Integrated Management

**DOI:** 10.3390/insects15040235

**Published:** 2024-03-28

**Authors:** Lance J. Meinke, Joseph L. Spencer

**Affiliations:** 1 Department of Entomology, University of Nebraska, Lincoln, NE 68583, USA; 2 Illinois Natural History Survey, Prairie Research Institute, University of Illinois, Champaign, IL 61820, USA; spencer1@illinois.edu

Species of the beetle genus *Diabrotica* (Coleoptera: Chrysomelidae) are native to North and South America, with their greatest diversity occurring in neotropical areas [1]. Little is known about the biology and ecology of many of the 400 described species, with current knowledge primarily limited to the small number of species that are pests in agricultural systems [1,2]. This Special Issue focuses primarily on key economically important pest species, i.e., *D. virgifera virgifera* LeConte, *D. speciosa* (Germar), *D. balteata* (LeConte), and *D. viridula* (F.).

The western corn rootworm, *D. v. virgifera*, is a key pest of grain maize in North America and the specific focus of many contributions to this Special Issue. The costs of managing this pest and the value of lost production annually exceed USD 2 billion in the U.S.A. This species was also accidentally introduced into Europe, where it is now an established maize pest [3]. The highly adaptable nature of this species has made management an ongoing challenge. Over time, this species has evolved resistance to active ingredients in four insecticide classes, annual crop rotation, and all commercially available rootworm-active Cry toxins (derived from the soil microbe *Bacillus thuringiensis)* expressed in *Bt*–maize hybrids in the U.S.A. [4,5,6].

The future success of *Diabrotica* pest management may depend on a more holistic view of management than that implemented in the past. This requires movement away from single-tactic approaches to a combination of tactics deployed within an integrated pest management framework [5]. This will include the conceptualization and development of new tactics that are based on an increased understanding of *Diabrotica* biology, physiology, ecology, and population dynamics [6,7,8,9,10].

This Special Issue provides original research and comprehensive reviews that summarize the current knowledge in key areas of *Diabrotica* biology, ecology, behavior, and management. The contributions include the following:

- An overview of the evolutionary history and host relationships of *Diabrotica* species, plus natural enemies of *Diabrotica* [1];
- The biology and management of *D. v. virgifera* in Europe [3] and *D. speciosa* (Germar), *D. balteata* (LeConte), and *D. viridula* (F.) in South America [2];
- Host–microbe relationships and aspects of chemical ecology that influence *D. v. virgifera* behavior and host plant resistance [9];
- The movement ecology of *D. v. virgifera* and its relation to management [6];
- The potential of RNAi technologies as components of *D. v. virgifera* management strategies [10];
- An overview of *D. v. virgifera* resistance to insecticides and plant-incorporated *Bt* traits in maize [4,5];
- Advances in *D. v. virgifera* monitoring/sampling technologies [7];
- An overview of the available computer models and modeling approaches to support research on the biology and management of *Diabrotica* species [8].

The history of field-evolved resistance is marked by repeated failures to appreciate the capabilities of pest insects. The western corn rootworm is a good example of this. Therefore, it is our hope that these publications, which refresh our current understanding of *Diabrotica*, will inform future research and efforts to develop novel and sustainable management tactics/strategies.
